# The Evolution of Skull Shape in *Boana faber* Clade: Unraveling Heterochrony's Influence

**DOI:** 10.1111/ede.70008

**Published:** 2025-05-13

**Authors:** Daniel de Abreu e Melo‐Moreira, Roberta Azeredo Murta‐Fonseca, Alessandra Silveira Machado, Ricardo Tadeu Lopes, Luciana Barreto Nascimento

**Affiliations:** ^1^ Programa de Pós‐graduação em Biodiversidade e Meio Ambiente Pontifícia Universidade Católica de Minas Gerais Belo Horizonte Brazil; ^2^ Laboratório de Zoologia, Campus do Pantanal, Universidade Federal de Mato Grosso do Sul Bairro Universitário Corumbá Brazil; ^3^ Instituto Alberto Luiz Coimbra de Pós‐Graduação e Pesquisa em Engenharia, Laboratório de Instrumentação Nuclear Universidade Federal do Rio de Janeiro Rio de Janeiro Brazil

**Keywords:** allometry, evo‐devo, geometric morphometrics, ontogeny, phylogenetic comparative methods

## Abstract

Variation in shape and size within a lineage, driven by developmental processes, plays a key role in diversification. Here, we explore the effects of allometry and heterochrony on the skull shape evolution during the post‐metamorphic period of species within the *Boana faber* clade, which vary considerably in body size. We analyzed 61 skulls of specimens belonging to eight species of the *Boana faber* clade, in addition to two outgroups, through 2D geometric morphometric analyses taken from CT‐Scan images. Our results demonstrated that skull shape is considerably impacted by the size, represented by centroid size, and this effect can be observed from ontogenetic and evolutionary perspectives. In this way, we accessed the ontogenetic trajectories of analysed species and, in light of the phylogenetic hypothesis of the clade, we discussed the observed variation based on the concept of heterochrony, suggesting that a peramorphic pattern has evolved in the group.

## Graphical abstract

The skull shape evolution of the *Boana faber* clade demonstrates a specific skull shape for each species, ontogenetic and evolutionary allometries, and a peramorphic tendency within the clade.

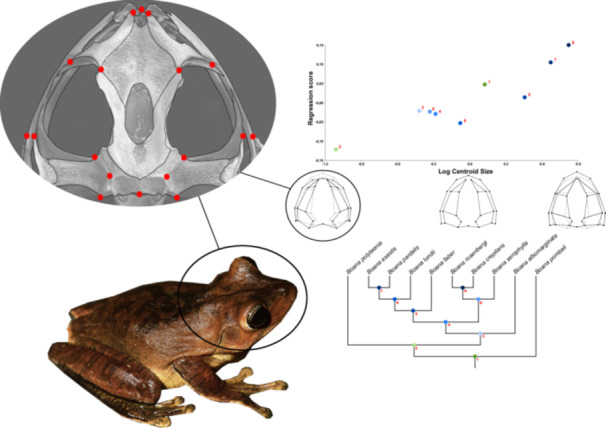

## 1 Introduction

Shape variation of an anatomical structure reflects how different evolutionary mechanisms can act on phenotypic selection (Fabre et al. 2020; Paluh et al. 2020; Bardua et al. 2021). Sometimes, size can affect shape and acts as a trigger for shape variation, a phenomenon called allometry, which can be detected on an intraspecific (static and ontogenetic allometries) and interspecific scale (evolutionary allometry) (Klingenberg 2016). One possible explanation for an allometric pattern is the heterochrony, which refers to an effect originated by subtle changes in the timing and/or rates of development of a given structure compared to their ancestral condition (Alberch et al. 1979; McNamara 2012; Ponssa and Candioti 2012; Dobreva et al. 2022). Thus, from an evolutionary point of view, heterochrony describes a relationship between ontogeny and phylogeny, since it is located at the interface of the causal‐reciprocal interrelations between the development and evolution of organisms (Klingenberg 1998; Fabrezi 2012; Ollonen et al. 2024). Although allometry focuses on the relationship between shape and size, and heterochrony concerns the relationship between shape and development, in species with a tendency to a continuous development period, such as amphibians, these concepts can overlap, as larger individuals also tend to be more developed (Fabrezi 2012; Dobreva et al. 2022).

Despite the confusing literature involving heterochrony and many disagreements (Gould 1977; Alberch et al. 1979; Reilly et al. 1997; Klingenberg 1998; Fabrezi 2012), generally two patterns of expression of the descendant organism's shape are recognized: paedomorphosis (underdevelopment compared to the ancestor) and peramorphosis (overdevelopment compared to the ancestor) (Alberch et al. 1979; McNamara 2012). Such patterns may originate from variations in relation to the ancestral condition of three complementary processes: the growth rate, the end of growth, and the onset of growth (Alberch et al. 1979). Thus, paedomorphosis can be expressed as deceleration/neoteny (lower growth rate), progenesis/hypomorphosis (premature end of the growth period), and post‐displacement (delay in the onset of growth), while peramorphosis can be expressed as acceleration (higher growth rate), hypermorphosis (extension of the growth period), and pre‐displacement (early onset of growth) (Gould 1977; Alberch et al. 1979; McNamara 2012).

Among terrestrial vertebrates, anurans are highlighted as models for studies related to morphological evolution, and this potential has been associated not only with the diversity of known species but also with variability in body size, a feature that, in these organisms, is linked to a prolonged growth period (Fabrezi et al. 2017; Duport‐Bru et al. 2019; Womack and Bell 2020). Recently, the application of modern techniques, such as geometric morphometrics and phylogenetic comparative methods, has been of great importance for understanding phenotypic evolution, revealing patterns of shape variation in different structures of the anurans, such as the skull (Duport‐Bru et al. 2019; Paluh et al. 2020; Bardua et al. 2021). Studies on this theme have demonstrated that anuran skull shape is not static during the post‐metamorphic period and that interspecific variation may be associated with the effects of allometry and heterochrony (e.g. Ponssa and Candioti 2012; Bardua et al. 2019, 2021; Duport‐Bru et al. 2019; Vučić et al. 2019). Furthermore, these shape variations represent a valuable source of information addressing functional, evolutionary, and taxonomic questions, which reinforces the need to explore this theme (Duport‐Bru et al. 2019; Paluh et al. 2020; Bardua et al. 2021).

The *Boana faber* clade (*sensu* Faivovich et al. 2005) is phylogenetically supported by molecular characters and, to date, includes ten species: *B. albomarginata* (Spix, 1824), *B. crepitans* (Wied‐Neuwied, 1824), *B. exastis* (Caramaschi and Rodrigues, 2003), *B. faber* (Wied‐Neuwied, 1820), *B. lundii* (Burmeister, 1856), *B. pardalis* (Spix, 1824), *B. pugnax* (Schmidt, 1857), *B. plantanera* La Marca, Escalona, Castellanos, Rojas‐Runjaic, Crawford, Señaris, Fouquet, Giaretta, and Castroviejo‐Fisher, 2021, *B. rosenbergi* (Boulenger, 1898), and *B. xerophylla* (Duméril and Bibron, 1841). The clade has recently been part of a phylogenetic study (Faivovich et al. 2021) and exhibits evident size differences between species, ranging from about 45 mm in *B. albomarginata* to 97 mm in *B. faber* (Escalona et al. 2021; see also Table S1 in Supporting information), making it an excellent model for studying the effects of allometry and heterochrony on skull shape diversity.

In the present study we investigated skull shape evolution of species from the *Boana faber* clade, applying the concepts of allometry and heterochrony to understand the observed morphological variations. Specifically, our goals were: to (1) explore skull shape variation within *Boana faber* clade, especially the (2) ontogenetic and (3) evolutionary allometry, and to (4) discuss the effect of allometry in the context of heterochrony.

## 2 Materials & Methods

### 2.1 Sampling

We analyzed material housed in the Herpetological Collection of Museu de Ciências Naturais of Pontifícia Universidade Católica de Minas Gerais (MCNAM ‐ Appendix 1), Amphibians Collection of Museu Nacional, Universidade Federal do Rio de Janeiro (MNRJ ‐ Appendix 1), and Amphibians Collection of Zoology and Botany Department, Universidade Estadual Paulista, São José do Rio Preto (DZSJRP ‐ Appendix 1). We analyzed 61 post‐metamorphic specimens belonging to 8 of the ten species of the *Boana faber* clade (*sensu* Faivovich et al. 2005): *Boana albomarginata* (*n* = 10), *B. crepitans* (*n* = 8), *B. exastis* (*n* = 2), *B. faber* (*n* = 10), *B. lundii* (*n* = 18), *B. pardalis* (*n* = 10), *B. rosenbergi* (*n* = 1), and *B. xerophylla* (*n* = 2). Furthermore, we included, as representatives of an outgroup, *B. polytaenia* (Cope 1870) (*n* = 1), of the *Boana pulchella* clade (*sensu* Faivovich et al. 2005), and *B. pombali* (Caramaschi, Pimenta and Feio 2004) (*n* = 1), of the *Boana semilineata* clade (*sensu* Faivovich et al. 2005).

Since anurans may exhibit sexual dimorphism in head/skull shape, even showing different allometric trajectories (e.g. Azeredo murta‐Fonseca et al. 2020; Melo‐Moreira et al. 2021), analyzing females and males together could introduce bias into our results. To avoid this, and considering that males are more abundant in collections and exhibit visible secondary sexual characteristics that help confirm their developmental stage, we chose to analyze only males. We acknowledge that this approach represents a limitation in the study, and further analyses should address this issue. In this way, we determined male sex based on the presence of vocal slits and protruding prepollical spines (Fabrezi et al. 2017). Additionally, for each species, we selected individuals encompassing the widest range of body sizes (snout‐vent length ‐ SVL) available in the collections (Table S1; Supporting information). To measure the SVL, we used a digital caliper with an accuracy of 0.01 mm. Moreover, here, we consider that SVL is a predictor for age, as demonstrated for several amphibians (Halliday and Verrell 1988; Duellman and Trueb 1994; Otero et al. 2017; Baraquet et al. 2021).

To obtain the images for subsequent analyses, we subjected the specimens to microcomputed tomography (microCT), using a high‐resolution microCT Scan Skyscan 1273/Bruker System, at Instituto Alberto Luiz Coimbra de Pós‐Graduação e Pesquisa de Engenharia (COPPE), Laboratório de Instrumentação Nuclear (LIN), Universidade Federal do Rio de Janeiro (UFRJ), Rio de Janeiro, Brasil. The parameters for image acquisition varied depending on the size of the specimen and are detailed in Table S2 (Supporting information). After acquisition, three‐dimensional (3D) images were reconstructed using the Feldkamp (FDK) algorithm (Feldkamp et al. 1984). After reconstruction, we used the software TpsDig 2 version 2.32 (Rohlf 2016) to measure skull length (SL) and skull width (SW) of each individual (Table S1; Supporting information).

### 2.2 Geometric Morphometrics

To characterize skull shape, we applied the geometric morphometrics (GM) method in a two‐dimensional (2D) approach. In this sense, we acquired photographs of the dorsal view of each skull using the software CTVox, version 2.7.0 (Bruker Corporation, Billerica, MA, USA). To evaluate error due to positioning 3D models to produce a 2D image and of landmarks digitization, we produced two images of each individual, followed by a duplication of each image, generating, in the end, four image files of each specimen analyzed.

**Figure 1:**
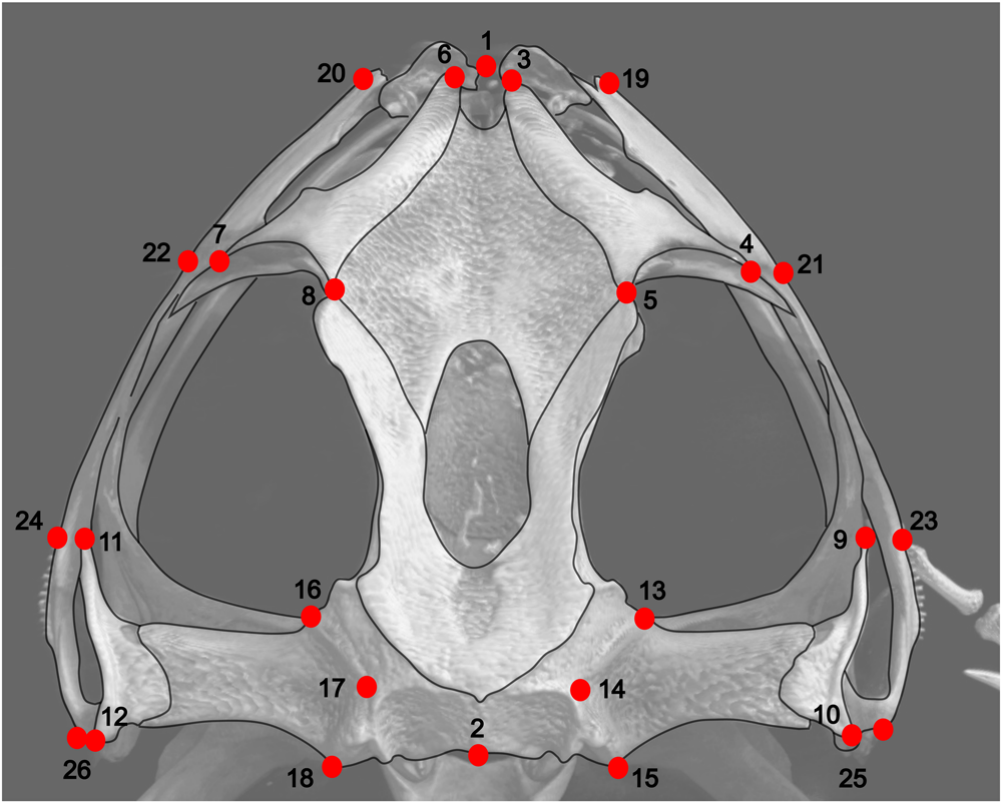
Configuration of landmarks in dorsal view (see Table 1), selected for the skull of the species analyzed. [Color figure can be viewed at wileyonlinelibrary.com]

To compile and convert images to be analyzed, we used the TPSUtil software, version 1.46 (Rohlf 2010). We digitized 26 landmarks in the dorsal view of the skull (Figure 1; Table 1) using the software TpsDig 2. Landmarks were selected based on their ability to represent the skull shape and be easily recognized and replicated across the entire data set (Souto et al. 2019). To minimize any methodological bias, all 2D images and landmark digitization were made by the same person (DAMM). The anatomical terminology used to describe landmarks were adapted from Trueb et al. (2000) and Sheil and Alamillo (2005).

**Table 1:** Landmarks used in the geometric morphometric analysis of the dorsal view of the skull of *Boana faber* clade species. Collateral points are listed in parentheses.

Landmark	Description
1	Most rostromedial point of the skull
2	Caudal end of the skull
3 (6)	Most rostral point of the nasal
4 (7)	Most caudolateral point of the nasal
5 (8)	Most caudomedial point of the nasal
9 (11)	Rostrolateral end of the squamosal
10 (12)	Caudolateral end of the squamosal
13 (16)	Craniomedial end of the otic capsule
14 (17)	Most medial point between points 13 and 15 (16 and 18)
15 (18)	Caudomedial end of the otic capsule
19 (20)	Edge of the jaw, parallel to point 3 (6)
21 (22)	Edge of the jaw, parallel to point 4 (7)
23 (24)	Edge of the jaw, parallel to point 9 (11)
25 (26)	Base of the squamosal

We used the software MorphoJ version 2.0 (Klingenberg 2011) to superimpose the landmarks through Orthogonal Superposition of Least Squares, or Generalized Procrustes Analysis (GPA) (Rohlf and Slice 1990), considering object symmetry. Structures with object symmetry are symmetric in itselves and, therefore, have an internal line or plane of symmetry, so that their left and right halves are mirror images of each other. Such configurations present two kinds of landmarks, paired and unpaired. In GM analyses, the symmetry should be considered to avoid statistical errors. Before superimposition, a reflected copy of each configuration is generated. Then, the paired landmarks of the reflected copies are relabeled so that each paired landmark obtains the label of its counterpart. The reflection first brings the landmarks to the opposite side, and the relabeling makes the arrangement of landmarks compatible with the original forms again. The Procrustes analysis includes the original and the mirrored configurations of a sample combined, and superimposes all of them simultaneously. After that, we have two shape componentes, the symmetric and the asymmetric. The symmetric component corresponds to the averages of original and reflected configurations (Klingenberg et al. 2002). In the GM analyses, whenever size was considered, it referred to the centroid size. The centroid of a configuration of landmarks is its centre of gravity, and its size is defined as the square root of the sum of the squared distances of all landmarks from their centroid (Bookstein 1991; Zelditch et al. 2004). Centroid size is commonly used as size measurement in GM analyzes since it is mathematically independent of shape (Zelditch et al. 2004).

### 2.3 Phylogenetic Comparative Methods

Phylogenetic Comparative Methods (PCM) were conducted based on the phylogenetic hypothesis of Faivovich et al. (2021), through which we extracted branch length values that were integrated into the Independent Contrast analysis (Felsenstein 1985; Maddison and FitzJohn 2015). The method assumes that characters evolve independently, through Brownian motion, with variations between rates (Felsenstein 1985). Independent Contrasts were used in the evolutionary allometry analysis.

### 2.4 Analyses

We performed a Procrustes Analysis of Variance (ANOVA) to test for error in positioning the skulls for photography and digitization of landmarks (Klingenberg and McIntyre 1998; Klingenberg et al. 2002; Klingenberg 2015). All analyses were performed with the symmetrical component of the dorsal view of the skull. The difference between two images of the same individual and two landmark digitizations of the same image were smaller than the differences between individuals (Table S3; Supporting information). We considered the error negligible and proceeded with further analyzes using the mean Procrustes coordinates of the four images of each individual.

For the ontogenetic allometry analysis, it is necessary to have a sample with the widest possible range of sizes for each species, therefore, species represented by only one or two individuals were removed from such analysis. The following data set was used: *Boana albomarginata* (*n* = 10), *B. crepitans* (*n* = 8), *B. faber* (*n* = 10), *B. lundii* (*n* = 18), and *B. pardalis* (*n* = 10). First, we performed a regression test of the Procrustes coordinates on the logarithm of the centroid size (Drake and Klingenberg 2008). We used a permutation test to determine the significance of the results. In this context, the permutation test uses the null hypothesis of complete independence between shape and size, simulating this null hypothesis by randomly reassigning observations for the shape to observations for the size, with 10 000 iterations for each test (Klingenberg et al. 2012; Klingenberg 2016). Ontogenetic allometry was tested for each species using the “pooled within subgroups” option, available in MorphoJ.

Evolutionary allometry analysis, the evolutionary change of shape that is associated with evolutionary change of size, was performed through a multivariate regression test using the independent contrasts of the Procrustes coordinates and the independent contrasts of the logarithm of the centroid size as shape and size variables, respectively (Klingenberg and Marugán‐Lobón 2013). In this approach, each species must be represented by only one configuration; therefore, for those species in which we had more than one specimen, we selected the individual with the largest centroid size among our sample. The following data set was included: eight individuals of the ingroup species ‐ *Boana albomarginata*, *B. crepitans*, *B. exastis*, *B. faber*, *B. lundii*, *B. pardalis*, *B. rosenbergi*, and *B. xerophylla*; and two outgroup representatives ‐ *B. polytaenia* and *B. pombali*.

To compare the allometric slopes and infer about the effect of heterochrony, we performed a multivariate analysis of variance (MANOVA), considering the logarithm of the centroid size, species, and the interaction between the parameters. To evaluate differences in allometric slopes, we performed a Pairwise test, considering the interaction between the Procrustes coordinates (shape) and logarithm of the centroid size (size) in each pair of species.

To explore shape variation within the clade, it is important to consider intraspecific variation, therefore, only species with *n* ≥ 8 were included in this approach, resulting in the same data set that was used for the ontogenetic allometry analysis. We first performed a Principal Component Analysis (PCA) of the data without size correction. Then, a second PCA was performed using the size‐corrected data (regression residuals). Finally, we performed a Canonical Variate Analysis (CVA) using the regression residuals. This analysis shows the greatest possible difference between groups (species being considered here as groups) in the smallest possible number of dimensions. This approach aims to test the shape distinction between species, complementing PCA.

All analyzes were performed in the software MorphoJ version 2.0 (Klingenberg 2011), with the exception of MANOVA and the Pairwise test, which were performed in the Geomorph 3.0.7 package (Adams and Otárola‐Castillo 2013) of the R platform (R Core Team 2018).

## 3 Results

In our sample, regarding SVL, the variation in each species was as follows: *B. albomarginata* (*n* = 10; 41.20 mm–52.68 mm); *B. crepitans* (*n* = 8; 52.52 mm–67.08 mm); *B. exastis* (*n* = 2; 85.57 mm–86.84 mm); *B. faber* (*n* = 10; 79.03 mm–95.93 mm); *B. lundii* (*n* = 18; 47.41 mm–67.96 mm); *B. pardalis* (*n* = 10; 49.83 mm–64.54 mm); *B. polytaenia* (*n* = 1; 24.24 mm); *B. pombali* (*n* = 1; 50.54 mm); *B. rosembergi* (*n* = 1; 80.29 mm); and *B. xerophylla* (*n* = 2; 48.71 mm–56.57 mm). The SVL, as well as SL and SW of each individual analyzed are presented in Table S1 (Supporting information).

**Figure 2:**
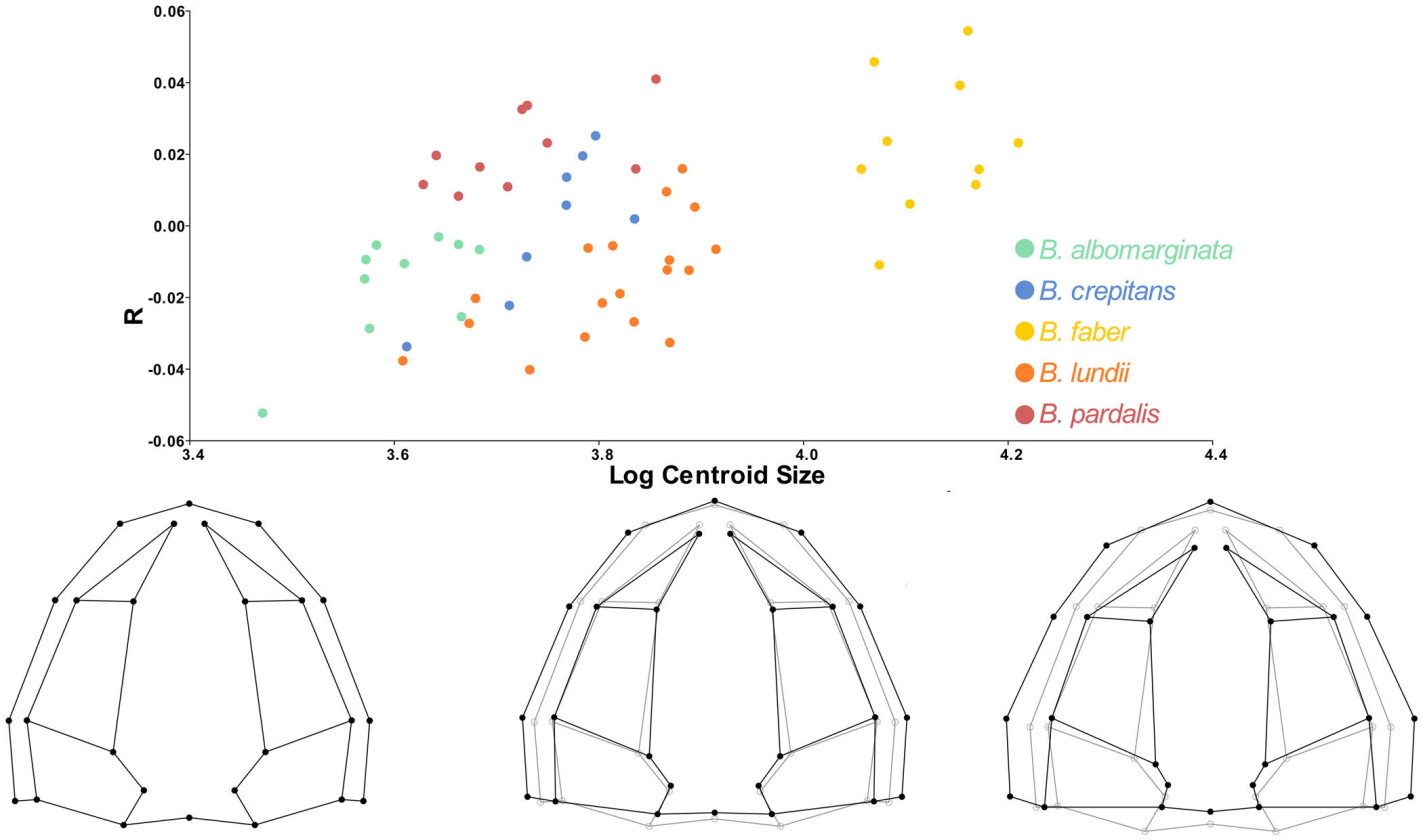
Regression analysis of the Procrustes coordinates of the dorsal view of the skull of species from the *Boana faber* clade by the logarithm of the centroid size. The settings at the bottom describe the variation of the X axis (centroid size), while the Y axis is related to the regression score (see Drake and Klingenberg 2008 for details). Light lines represent the average shape of the sample, while dark lines represent the predicted shape for centroid sizes 0.0, 0.5, and 1.0. [Color figure can be viewed at wileyonlinelibrary.com]

**Figure 3:**
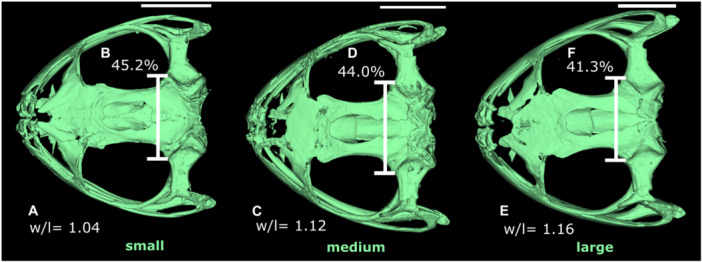
Skull shape changes in *Boana albomarginata* associated with size increase. Smaller individual is represented by MCNAM13607 (SVL = 41.20 mm), medium individual by MCNAM13915 (SVL = 47.28 mm), and larger individual by MCNAM2230 (SVL = 52.68 mm). Legend. Proportion between width and length in (A) smaller, (C) medium, and (E) larger individuals; distance between landmarks 13 and 16 relative to skull width in (B) smaller, (D) medium, and (F) larger individual. This figure illustrates that, as size increases, there is a tendency for the skull to become wider, however, the distance between the craniomedial ends of the optic capsules (vertical bars) becomes proportionally smaller (b, d, f). [Color figure can be viewed at wileyonlinelibrary.com]

**Figure 4:**
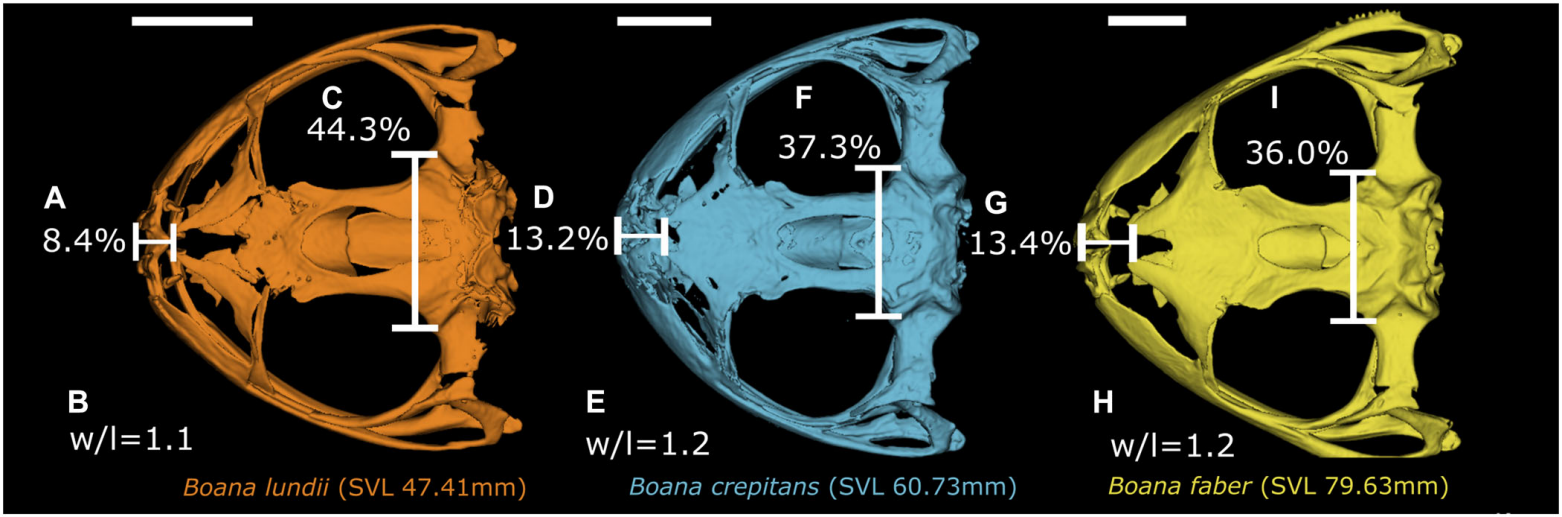
Skull shape changes in *Boana faber* clade associated with size increase. Smaller individual is represented by *Boana lundii* MCNAM9543 (SVL = 47.41 mm), medium individual by *Boana crepitans* MCNAM6384 (SVL = 60.73 mm), and larger individual by Boana faber MCNAM16881 (SVL = 79.63 mm). Legend. Snout length relative to skull length in (A) *B. lundii*, (D) *B. crepitans*, and (G) *B. faber*; proportion between width and length in (B) *B. lundii*, (E) *B. crepitans*, and (H) *B. faber*; distance between landmarks 13 and 16 relative to skull width in (C) *B. lundii*, (F) *B. crepitans*, and (I) *B. faber*. The figure illustrates the same shape trend associated with size increase observed in Figure 3, along with a tendency for the snout to become proportionally larger. [Color figure can be viewed at wileyonlinelibrary.com]

Through the permutation test, considering the null hypothesis of isometric growth, we found statistical significance for ontogenetic allometry in the dorsal view of the skull (*p* < 0.05). In the *Boana faber* clade, 7.9% of shape variation is explained by size (Figure 2). Larger centroid sizes are related to a wider and rostrally projected skull, with a shortening of the caudal region, in addition to a tendency for the nasals to move caudally (Figures 3 and 4). Figure 3 illustrates such shape changes at an intraspecific level throughout the ontogeny of *B. albomarginata*. Smaller individuals exhibit a greater distance between landmarks 13 and 16, which represent the craniomedial end of the otic capsule, as well as a smaller ratio of skull width to length. A similar shape trend can be observed in an interspecific context (Figure 4), along with an enlargement of the snout length associated with an increase in size. Overall, we found that the skull shape of larger individuals of smaller species tends to be similar to the skull shape of smaller individuals of larger species.

**Figure 5:**
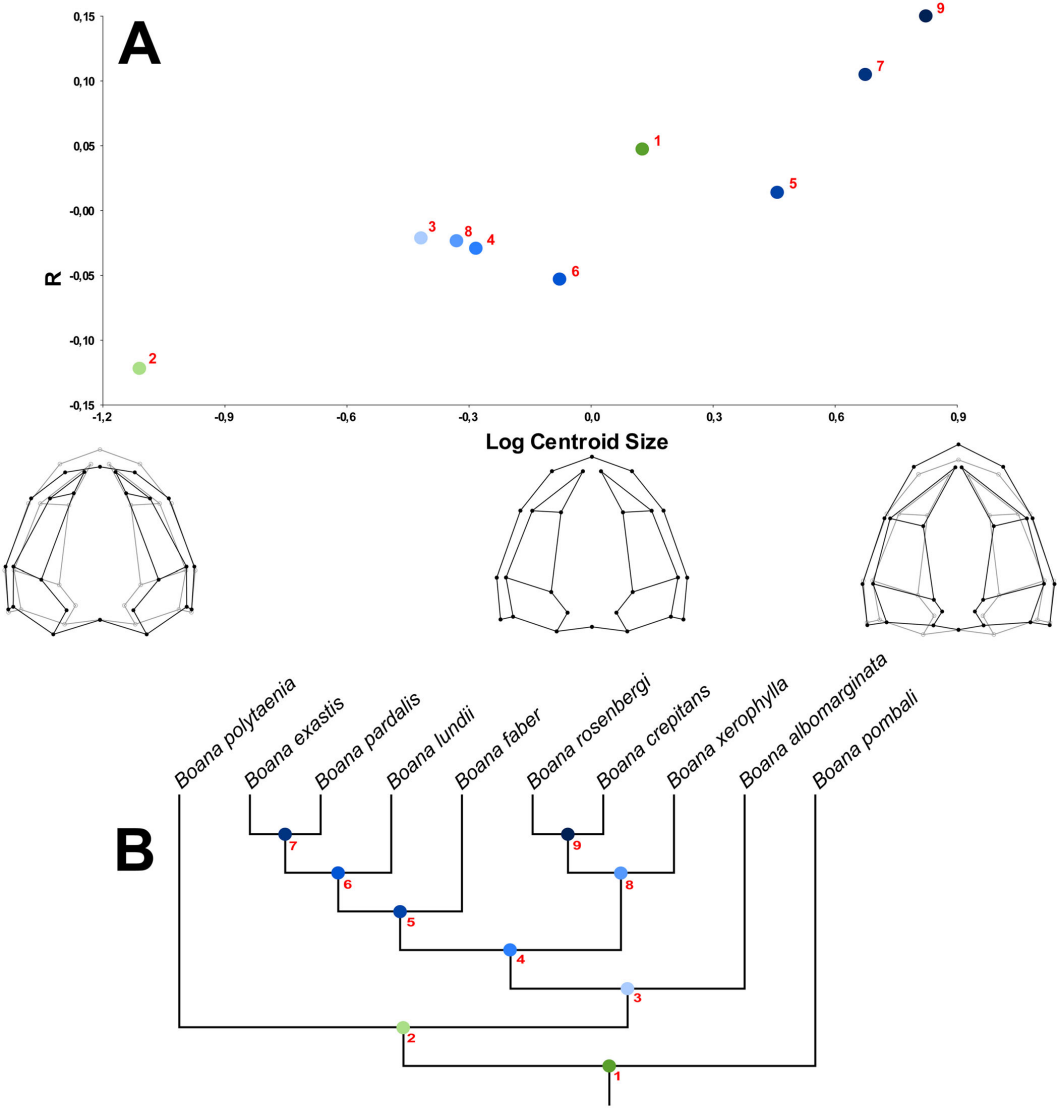
(A). Multivariate regression test of independent contrasts of Procrustes coordinates of the dorsal view of the skull of species from the *Boana faber* clade (*Boana albomarginata*, *B. crepitans*, *B. exastis*, *B. faber*, *B. lundii*, *B. pardalis*, *B. rosenbergi* and *B. xerophylla*) and outgroup representatives (*B. polytaenia*, *B. pombali*) by independent contrasts of the logarithm of the centroid size. The settings at the bottom describe the variation of the X axis (centroid size). Light lines represent the average shape of the sample, while dark lines represent the predicted shape for logarithm of the centroid sizes ‐1.0, 0.0, and 0.9. The numbered and colored points on the graph represent the nodes of the phylogeny (B), which corresponds to a pruned phylogenetic hypothesis of Faivovich et al. (2021), showing only the analyzed taxa. [Color figure can be viewed at wileyonlinelibrary.com]

Evolutionary allometry also showed statistical significance (*p* < 0.05), with 46.2% of the shape variation being related to size (Figure 5). Larger skulls are longer, with a shorter caudal region, and more elongated nasals.

The MANOVA demonstrated a significant result for centroid size and species (*p* < 0.05) (Table S4; Supporting information), which suggests statistically significant allometry in the data set and statistically significant shape differences between species. There was, however, no interaction between these factors, indicating that the allometric slopes are the same in the species of *Boana faber* clade. The pairwise test confirmed this result, showing no difference in the ontogenetic trajectory between all pairs of species (Table S5; Supporting information). This result suggests that variation is influenced by the effect of heterochrony.

**Figure 6:**
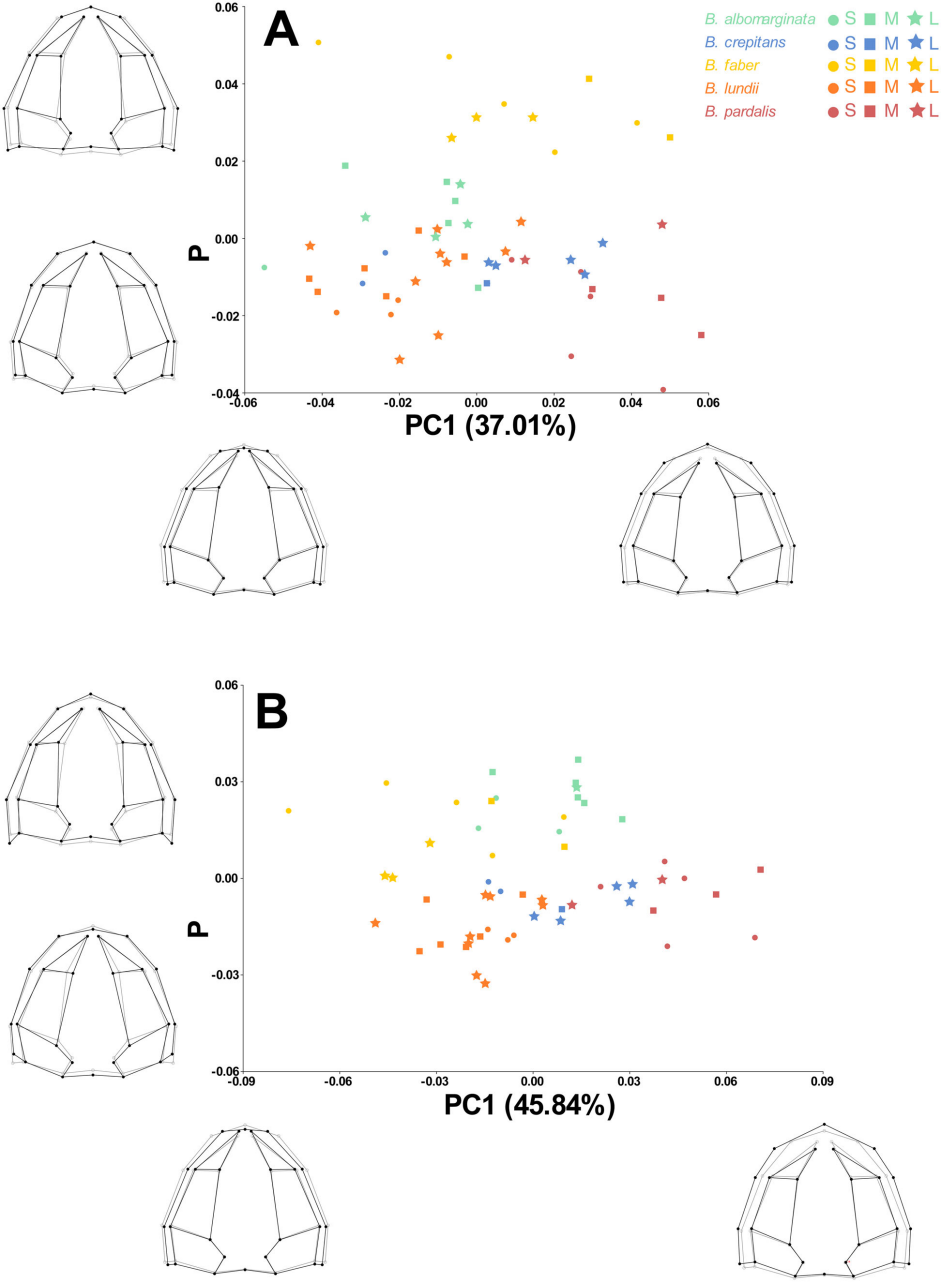
Principal Component Analysis of the dorsal view of the skull of species from the *Boana faber* clade (PC1 vs. PC2) with (A) uncorrected size, and (B) corrected size. The gray lines represent the average skull shape, while the black lines represent the settings for the maximum and minimum values on each axis of the PC. Circles represent small specimens (S), squares represent medium specimens (M), and stars represent large specimens (L) within each species. For *B. albomarginata*, individuals with centroid size between 32.1 and 34.7 were considered small; 34.8–37.2 were considered medium; and 37.3–39.7 were considered large. For *B. crepitans*, those individuals between 37.0 and 40.1 were considered small; 40.2–43.2 were considered medium; and 43.3‐46.2 were considered large. For *B. faber*, specimens between 57.7 and 60.9 were considered small; 61.0–64.1 were considered medium; and 64.2‐67.3 were considered large. For *B. lundii*, specimens between 36.9 and 41.3 were considered small; 41.4–45.7 were considered medium; and 45.8‐50.1 were considered large. Finally, for *B. pardalis*, specimens between 37.6 and 40.8 were considered small; 40.9–44.0 were considered medium; and 44.1–47.2 were considered large. [Color figure can be viewed at wileyonlinelibrary.com]

**Figure 7:**
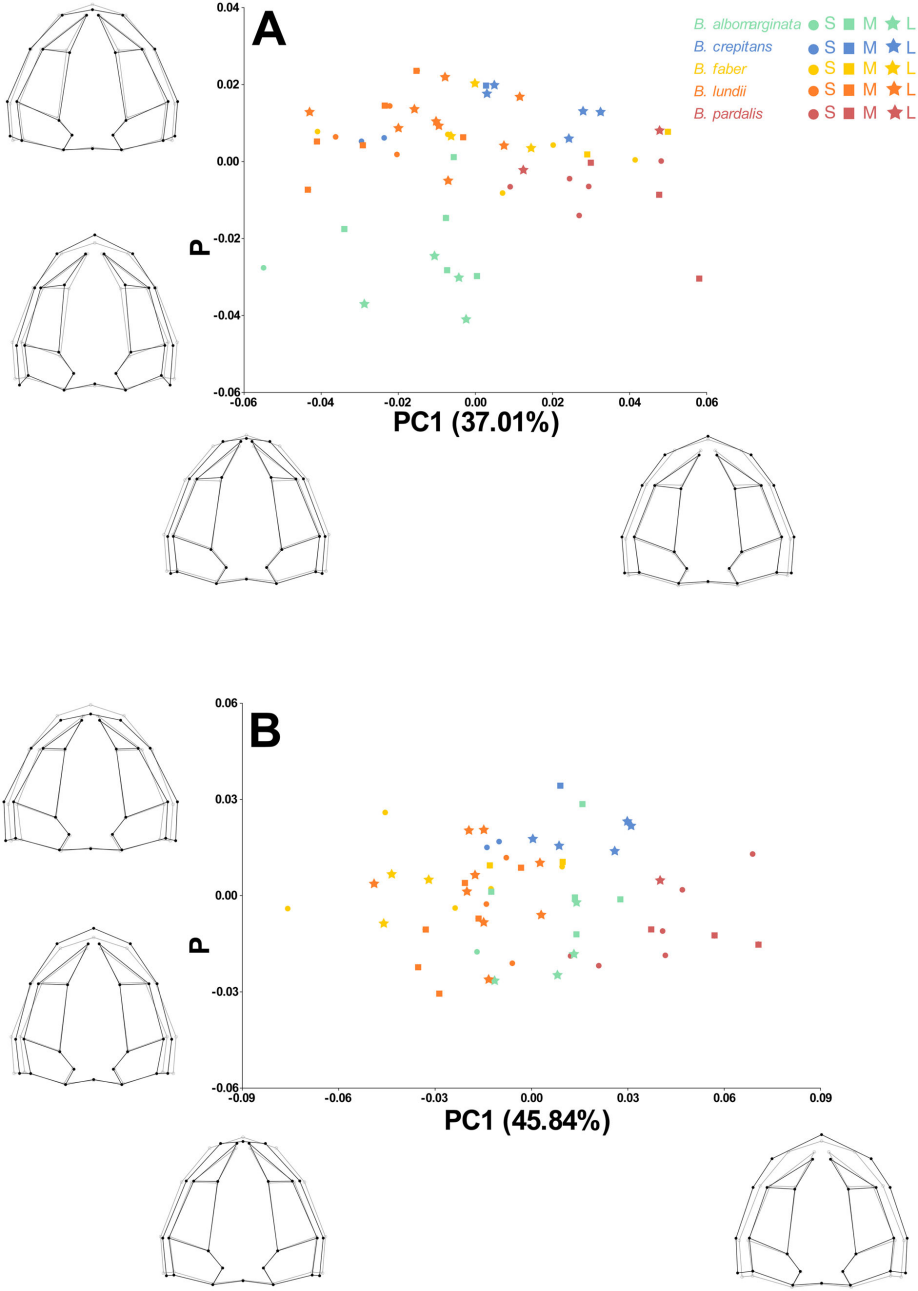
Principal Component Analysis of the dorsal view of the skull of species from the *Boana faber* clade (PC1 vs. PC3) with (A) uncorrected size, and (B) corrected size. The gray lines represent the average skull shape, while the black lines represent the settings for the maximum and minimum values on each axis of the PC. Circles represent small specimens (S), squares represent medium specimens (M), and stars represent large specimens (L) within each species. For *B. albomarginata*, individuals with centroid size between 32.1 and 34.7 were considered small; 34.8–37.2 were considered medium; and 37.3–39.7 were considered large. For *B. crepitans*, those individuals between 37.0 and 40.1 were considered small; 40.2–43.2 were considered medium; and 43.3–46.2 were considered large. For *B. faber*, specimens between 57.7 and 60.9 were considered small; 61.0–64.1 were considered medium; and 64.2–67.3 were considered large. For *B. lundii*, specimens between 36.9 and 41.3 were considered small; 41.4–45.7 were considered medium; and 45.8–50.1 were considered large. Finally, for *B. pardalis*, specimens between 37.6 and 40.8 were considered small; 40.9–44.0 were considered medium; and 44.1–47.2 were considered large. [Color figure can be viewed at wileyonlinelibrary.com]

Through PCA, we identified that 68.9% of shape variation is represented by the first three axes. The first axis (37.0%) shows, in the extreme with positive values, longer and wider skulls, with shorter nasaal bones (Figure 6A). In this axis, it is possible to distinguish *Boana pardalis* from *B. lundii* and *B. abomarginata*, while *B. crepitans* and *B. faber* are distributed more homogeneously. The PC2 (19.4%) shows positive values associated with shorter and wider skulls, with slightly longer nasals (Figure 6A). In this axis, it is possible to distinguish *B. faber* from all other species. The scatterplot depicting axes 1 versus 2 does not show a tendency of larger individuals of smaller species to be closer to smaller individuals of larger species, which indicates that it is not only heterochrony that is shaping skull morphology. Instead, the image present well‐defined groups for the species, although *B. crepitans* overlaps the morphospace of *B. lundii* and *B. pardalis*, in addition to a small overlap between *B. albomarginata* with *B. lundii* and *B. crepitans*. The third axis (12.4%) presents positive values associated with shorter and wider skulls, with slightly more dilated nasals (Figure 7A). Once more, there was no tendency of individuals to group by size. In this axis, *B. albomarginata* is clearly apart from all others. In summary, the only species that cannot be distinguished from the others based on the combination of the first three axes is *B. crepitans*.

After size correction (residuals from the regression), the first three axes of the PCA explained 74.0% of the shape variation (PC1–45.8%; PC2–15.7%; PC3–12.4%). In this approach, the positive values of PC1 correspond to variations similar to those observed when there was no size correction (Figure 6B). The PC2 axis has positive values associated with narrower skulls and narrower nasal passages, opposed to what was observed in the raw data (Figure 6B). The PC1 versus PC2 scatterplot, once again, presents well‐defined groups considering species, not size, now with a small overlap between *B. albomarginata* and *B. faber*, and again with *B. crepitans* overlapping the morphospace of *B. lundii* and *B. pardalis*. In PC3, positive values are associated with shorter skulls and nasals when compared to such axes in the raw data PCA (Figure 7B). On this axis, *B. albomarginata* and *B. faber* are apart from each other, as well as between *B. crepitans* and *B. pardalis*. Considering the size corrected PCA, the only pair of species that can not be fully distinguished from each other based on the combination of the first three axes are *B. crepitans* and *B. lundii*.

**Figure 8:**
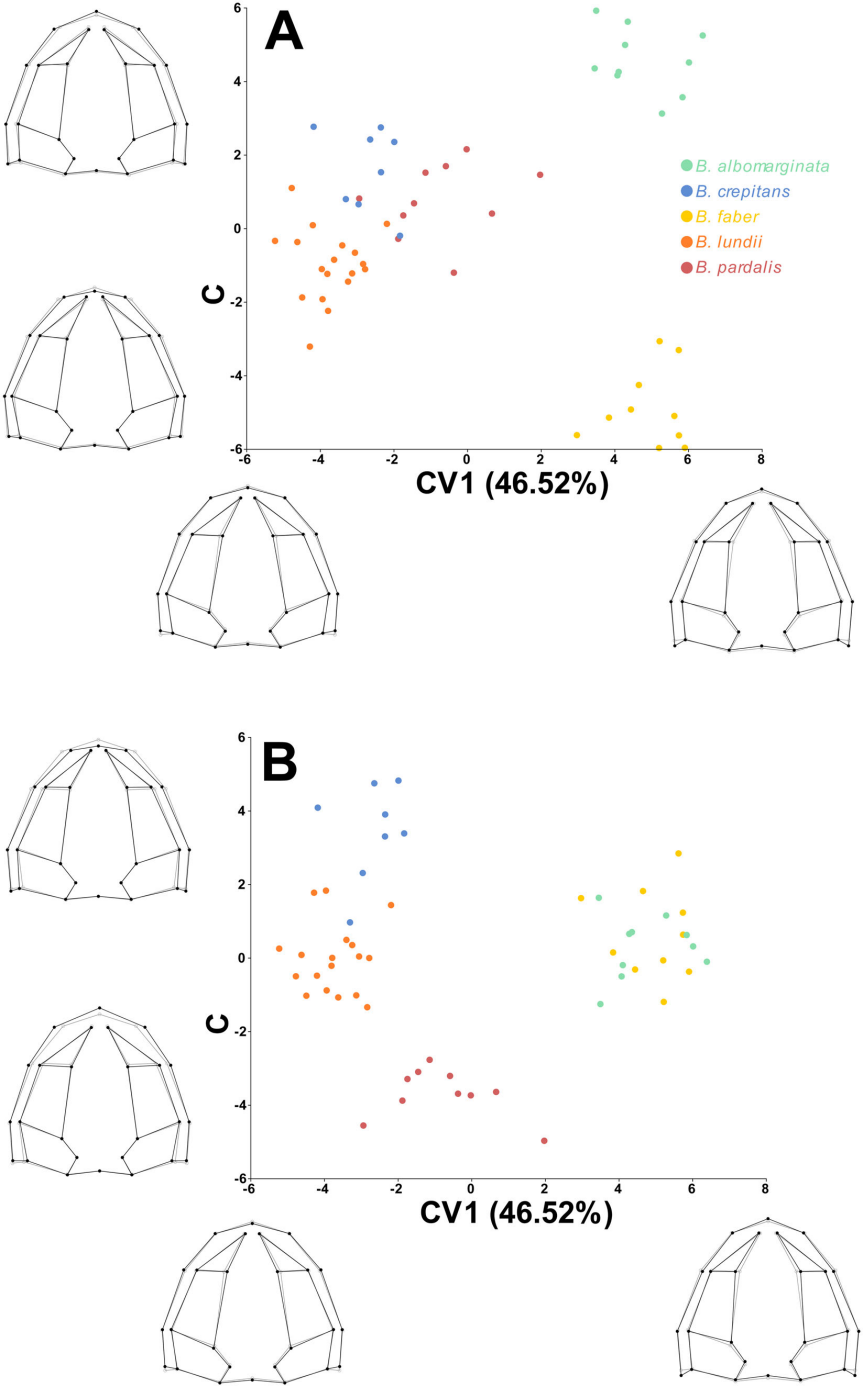
Canonical analysis of the dorsal view of the skull of species from the *Boana faber* clade. (A) CV1 versus CV2; (B) CV1 versus CV3. The gray lines represent the average skull shape, while the black lines represent the settings for the maximum and minimum values on each axis. [Color figure can be viewed at wileyonlinelibrary.com]

The CVA confirmed the tendency of species to group among themselves, showing all groups well delimited, with significant values for both Procrustes and Mahalanobis distances (Tables S6 to S9; Supporting information). The first three canonical variables (CV) summarize 89.8% of the shape variation (Figure 8). The CV1 explains 46.5% of shape variation, with positive values associated with longer and narrower skulls, with narrower nasals. The second canonical axis explains 29.3% and the positive values are associated with longer and narrower skulls, and shorter nasals, more medially oriented. Finally, the third canonical axis explains 13.9% of the shape variation, with positive values associated with shorter skulls and nasals (Figure 8). In the CV1 versus CV2 scatterplot (Figure 8A) all species are well grouped and delimited, with the exception of *B. crepitans*, which occupies an intermediate position between *B. lundii* and *B. pardalis*. In the CV1 versus CV3 (Figure 8B), however, the species *B. crepitans*, *B. lundii* and *B. pardalis* are well delimited.

## 4 Discussion

For those interested in evolution, studies that explore phenotypic diversity and the factors acting as selective pressures generating this diversity in shape are a central focus of research. In this context, heterochrony also plays an important role in shaping morphological diversity, as small changes in the timing and/or rates of development can lead to significant morphological changes, such as the allometric effect (Gould 1977; Alberch et al. 1979; Fabrezi 2012; Ollonen et al. 2024). In the present study we identified skull shape variation among post‐metamorphic male specimens in the *Boana faber* clade, and verified the effects of allometry in the ontogenetic and evolutionary perspectives, identifying a heterochronic pattern.

Even though the anuran skull shape has a conserved bauplan (Schoch 2014), through allometry analyses we verified that size represents an important driver of shape variation throughout the post‐metamorphic ontogeny of species from the *B. faber* clade. During this period, as the size increases, the skull of such species tends to lengthen in the most rostral portion (dermatocranium) accompanied by shortening of the most caudal region (neurocranium). These variations between the proportions of the dermatocranium and neurocranium are directly associated with skull size from both an ontogenetic and evolutionary perspective (see Figures 3 and 4) and corroborates what previous studies reported for other groups of anurans (Ponssa and Candioti 2012; Duport‐Bru et al. 2019; Azeredo murta‐Fonseca et al. 2020; Bardua et al. 2021). Moreover, on a more comprehensive phylogenetic scale, such ontogenetic development is a frequent pattern in vertebrates (Hanken and Thorogood 1993; Depew and Simpson 2006; Young et al. 2014), having been demonstrated in more details for squamate reptiles (e.g. Murta‐Fonseca and Fernandes 2016; Ollonen et al. 2024).

Many studies have shown that heterochrony may also represent a driver for the process of morphological diversification, leading to patterns such as allometry (Ponssa and Candioti 2012; Ivanović and Arntzen 2018; Duport‐Bru et al. 2019; Paluh et al. 2020; Bardua et al. 2021; Dobreva et al. 2022). Therefore, comparing the allometric trajectories of species throughout ontogeny allows us to investigate the heterochronic effect, identifying patterns and discussing associated processes (Paluh et al. 2020). Thus, considering that the slopes of the allometric trajectories of the species in the *B. faber* clade do not differ among themselves and that larger individuals of the smaller species have skulls resembling smaller individuals of larger species, we could infer that there are heterochronic changes in these structures along the evolution of the lineage.

*Boana faber* is a species larger than the other taxa of the *B. faber* clade (see Table S1; Supporting information), with the smaller individuals of such taxon presenting skull shape close to that of the larger individuals of the other species, a pattern also seen when comparing other species with different sizes analyzed herein (Figure 2). In studies of heterochrony, one can not state the pattern or determine the direction of heterochronic changes without information about the ontogeny of ancestor and descendant, the allometric trajectory, and a phylogenetic hypothesis (Reilly et al. 1997; Fabrezi 2012). The use of size as a metric for estimating age is common in anurans (e.g. Halliday and Verrell 1988; Ponssa and Candioti 2012; Duport‐Bru et al. 2019; Azeredo murta‐Fonseca et al. 2020), although it remains a subject of debate. While Yeh (2002b) highlighted the low accuracy of SVL as a proxy for age in pipoid frogs, Baraquet et al. (2021) demonstrated that, in *Boana pulchella* (Duméril and Bibron, 1841), SVL serves as a reliable predictor of age and, consequently, of ontogenetic stage. Here, we follow Baraquet et al. (2021) in using SVL as a proxy for age, considering that their study tested this relationship in a phylogenetically close species. However, we acknowledge that this approach is not universally accepted and that studies using skeletochronology in *B*. gr. *faber* would be a crucial step toward a better understanding of heterochrony in the group. In this way, we considered the three requirements, and our results point to a probable ancestor of the *B. faber* clade with a skull smaller than those of the extant species within such group (Figure 5), suggesting that a peramorphic pattern has evolved. This pattern is particularly evident in the allometric trajectory of *B. albomarginata*, the smallest and most basal species in the group, and *B. faber*, the largest species in the group (Figure 2). However, since we do not have explicit time measurements for the analyzed species, we can not determine whether the variation is the product of a developmental acceleration or represents a slow but prolonged development, which makes necessary for future research to focus on the temporal factor of development (Baraquet et al. 2021; Peng et al. 2022).

While we found allometry, originating from the effect of heterochrony, to be a strong factor generating the diversity of skull shapes in the *Boana faber* clade, this factor alone is not sufficient to explain all the shape variation observed. Skull shape allows for the distinction of all species from one another (Figures 6, 7, and 8) without necessarily being related to size. Thus, it's important to consider that aspects such as diet, habitat use, and locomotion are often associated with shape variation, even though the variation caused by such factors must occur within the limits that development allows (Emerson 1985; Vidal‐García and Scott Keogh 2017; Duport‐Bru et al. 2019; Fratani et al. 2020; Azeredo murta‐Fonseca et al. 2020; Paluh et al. 2020; Melo‐Moreira et al. 2021; Reis et al. 2024). In this sense, we can infer that the geometric patterns observed among the analyzed species may be the product of intrinsic factors (e.g., hormonal signals, age) and extrinsic factors (e.g., seasonality, food availability), but are not necessarily associated with any single factor, which underlies the complexity of evolutionary processes.

Although we did not include females in this study, it is important to keep in mind that possible sexual dimorphism would have the potential to alter the results and, consequently, the way in which we interpret variation (e.g. Azeredo murta‐Fonseca et al. 2020; Melo‐Moreira et al. 2021). It is possible, for example, that conspecific females and males present different allometric trajectories and this would have a significant effect on the allometric trajectory of the species as a whole (Badyaev 2002). In this way, Azeredo murta‐Fonseca et al. (2020) demonstrated how the allometric trajectory of females and males of *Nyctimantis brunoi* (Miranda‐Ribeiro, 1920) varies from an ontogenetic perspective, what could indicate different heterochronic patterns within the species, although it was not explored by the authors. In recent years, heterochrony has been extensively explored (Fabrezi 2012; McNamara 2012; Dobreva et al. 2022), however, there are still few studies that have sought to understand its effect on sexual dimorphism (e.g. Badyaev 2002; Le Galliard et al. 2006; Zhang et al. 2012).

Overall, our results allowed us to conclude that skull shape of the species of *B. faber* clade does not remain static after metamorphosis, with allometry acting on morphological variation within the group both from ontogenetic and evolutionary perspectives, contributing to a field of study that still has scarce literature. Furthermore, we found that the allometry observed in the group results from heterochronic processes, leading to a peramorphic pattern. We also infer that there are other factors molding shape diversity among the group and that a comprehensive study must include natural history aspects, as much as other physiological and developmental processes. Yeh (2002a), when analyzing the effect of miniaturization on the skull of a group of anurans, demonstrates how the nervous and sensory systems are limiting for the architecture of this structure. Thus, we suggest that some of the gaps that could be addressed in future studies to enrich this discussion include incorporating ecological, physiological, and developmental aspects, as well as morphological constraints, in addition to including females and investigating heterochrony in an intraspecific context.

## Conflicts of Interest

The authors declare no conflicts of interest.

## Supporting information

Supporting information.

## Data Availability

The authors have nothing to report.

## 1

**Appendix 1:** Specimens examined.

**COUNTRY:** Locality

***Boana albomarginata*** **(n** = **10). BRAZIL:** BAHIA: Itabuna MCNAM15841; Wenceslau Guimarães MCNAM2230. ESPÍRITO SANTO: Mimoso do Sul MCNAM13915; Praia das Neves MCNAM13607. MINAS GERAIS: Conceição do Mato Dentro MCNAM20674; São Francisco da Glória MCNAM14696; São Gonçalo do Rio Preto MCNAM3750; São José do Mantimento MCNAM11223; Serro MCNAM18974. SÃO PAULO: Cubatão MCNAM17675.

***Boana crepitans*** **(n** = **8). BRAZIL:** MINAS GERAIS: Açucena MCNAM14889, MCNAM6384; Conceição do Mato Dentro MCNAM18471; Grão Mogol MCNAM21084; Guanhães MCNAM1063; Mutum MCNAM7938; Porteirinha MCNAM173; Resplendor MCNAM1493.

***B. exastis*** **(n** = **2). BRAZIL:** BAHIA: Fazenda Capitão, Itacaré MNRJ35324, MNRJ35325.

***Boana faber*** **(n** = **10). BRAZIL:** MINAS GERAIS: Caeté MCNAM17593, MCNAM17595; Conceição do Mato Dentro MCNAM10762, MCNAM17006; Florestal MCNAM9510; Frei Inocêncio MCNAM10627; Itabira MCNAM057, MCNAM16881; São Domingos da Prata MCNAM1018. RIO DE JANEIRO: Nova Friburgo MCNAM6275.

***Boana lundii*** **(n** = **18). BRAZIL:** MINAS GERAIS: Araguari MCNAM7780; Belo Horizonte MCNAM11894, MCNAM14017; Bonfim MCNAM8606; Brumadinho MCNAM13010; Divinópolis MCNAM9543, MCNAM12952. Florestal MCNAM9513; Grão Mogol MCNAM6710, MCNAM7129, MCNAM9378, MCNAM9389, MCNAM9395, MCNAM16133; Ouro Preto MCNAM4112; Paracatu MCNAM8007; Uberlândia MCNAM1617; Unaí MCNAM8800.

***Boana pardalis*** **(n** = **10). BRAZIL:** MINAS GERAIS: Açucena MCNAM14891; João Monlevade MCNAM19022; Mutum MCNAM7931; Ouro Preto MCNAM1836; Paula Cândido MCNAM13532; Santa Maria do Salto MCNAM4026; São Francisco da Glória MCNAM14690; São José do Mantimento MCNAM11583, MCNAM11598. RIO DE JANEIRO: Itaverava MCNAM13544.

***Boana polytaenia*** **(n** = **1). BRAZIL:** MINAS GERAIS: Nova Lima MCNAM403.

***Boana pombali*** **(n** = **1). BRAZIL:** BAHIA: Itabuna MCNAM15858.

***Boana rosenbergi*** **(n** = **1). ECUADOR:** ESMERALDAS: Esmeraldas DZSJRP548.

***Boana xerophylla*** **(n** = **2). SURINAME:** MONTANHAS LELY: Sipaliwini MNRJ92500, MNRJ92501.
