# Supplementary material for: The Evolution of Skull Shape in Boana faber Clade: Unraveling Heterochrony's Influence
Source: Evol Dev. 2025 May 13;27(2):e70008. doi: 10.1111/ede.70008 (PMC12070859; doi:10.1111/ede.70008)
Supplement: Supplementary file 1 — Supporting information. [file EDE-27-e70008-s001.pdf]

**Supporting information**

**Table S1:** Morphometric data of the analyzed individuals. All measurements are in millimeters (mm). Legend: SVL - Snout-vent length; SL - Skull length; SW - Skull width.

| Species | Specimen | SVL | SL | SW |
|---|---|---|---|---|
| *Boana albomarginata* | MCNAM2230 | 52.68 | 15.74 | 16.68 |
| *Boana albomarginata* | MCNAM3750 | 48.35 | 14.67 | 15.70 |
| *Boana albomarginata* | MCNAM11223 | 49.95 | 14.39 | 15.44 |
| *Boana albomarginata* | MCNAM13607 | 41.20 | 12.58 | 13.00 |
| *Boana albomarginata* | MCNAM13915 | 47.28 | 14.37 | 15.59 |
| *Boana albomarginata* | MCNAM14696 | 51.22 | 15.47 | 16.50 |
| *Boana albomarginata* | MCNAM15841 | 49.81 | 15.13 | 15.78 |
| *Boana albomarginata* | MCNAM17675 | 45.21 | 13.83 | 16.23 |
| *Boana albomarginata* | MCNAM18974 | 46.13 | 13.55 | 15.64 |
| *Boana albomarginata* | MCNAM20674 | 51.03 | 14.26 | 16.82 |
| *Boana crepitans* | MCNAM173 | 67.08 | 18.46 | 20.81 |
| *Boana crepitans* | MCNAM1063 | 60.02 | 16.92 | 20.29 |
| *Boana crepitans* | MCNAM1493 | 58.96 | 16.78 | 19.55 |
| *Boana crepitans* | MCNAM6384 | 60.73 | 16.48 | 20.14 |
| *Boana crepitans* | MCNAM7938 | 57.94 | 16.50 | 19.74 |
| *Boana crepitans* | MCNAM14889 | 59.74 | 16.37 | 18.30 |
| *Boana crepitans* | MCNAM18471 | 52.52 | 14.06 | 15.45 |
| *Boana crepitans* | MCNAM21084 | 56.73 | 15.45 | 18.17 |
| *Boana exastis* | MNRJ35324 | 85.57 | 24.09 | 25.39 |
| *Boana exastis* | MNRJ35325 | 86.84 | 23.87 | 24.71 |
| *Boana faber* | MCNAM057 | 90.99 | 26.46 | 29.28 |
| *Boana faber* | MCNAM1018 | 91.32 | 24.43 | 28.68 |
| *Boana faber* | MCNAM6275 | 87.13 | 26.00 | 30.32 |
| *Boana faber* | MCNAM9510 | 79.82 | 22.44 | 26.95 |
| *Boana faber* | MCNAM10627 | 80.35 | 21.99 | 26.69 |
| *Boana faber* | MCNAM10762 | 90.07 | 26.77 | 31.29 |

| | | | | |
|---|---|---|---|---|
| *Boana faber* | MCNAM16881 | 79.63 | 23.77 | 26.72 |
| *Boana faber* | MCNAM17006 | 79.03 | 21.51 | 25.27 |
| *Boana faber* | MCNAM17593 | 93.10 | 22.69 | 27.46 |
| *Boana faber* | MCNAM17595 | 95.93 | 24.85 | 29.56 |
| *Boana lundii* | MCNAM1617 | 55.04 | 15.27 | 17.09 |
| *Boana lundii* | MCNAM4112 | 52.72 | 15.89 | 16.94 |
| *Boana lundii* | MCNAM6710 | 62.05 | 18.12 | 20.11 |
| *Boana lundii* | MCNAM7129 | 64.29 | 18.81 | 21.14 |
| *Boana lundii* | MCNAM7780 | 58.11 | 17.43 | 19.40 |
| *Boana lundii* | MCNAM8007 | 64.88 | 19.87 | 21.95 |
| *Boana lundii* | MCNAM8606 | 58.6 | 17.50 | 18.85 |
| *Boana lundii* | MCNAM8800 | 65.36 | 18.46 | 21.60 |
| *Boana lundii* | MCNAM9378 | 61.08 | 17.57 | 20.21 |
| *Boana lundii* | MCNAM9389 | 68.73 | 18.96 | 22.33 |
| *Boana lundii* | MCNAM9395 | 60.18 | 17.96 | 19.72 |
| *Boana lundii* | MCNAM9513 | 60.78 | 17.96 | 18.55 |
| *Boana lundii* | MCNAM9543 | 47.41 | 14.45 | 15.63 |
| *Boana lundii* | MCNAM11894 | 64.48 | 19.39 | 20.84 |
| *Boana lundii* | MCNAM12952 | 65.06 | 18.84 | 20.84 |
| *Boana lundii* | MCNAM13010 | 61.11 | 18.50 | 21.00 |
| *Boana lundii* | MCNAM14017 | 67.96 | 17.90 | 22.10 |
| *Boana lundii* | MCNAM16133 | 56.45 | 16.53 | 18.00 |
| *Boana pardalis* | MCNAM7931 | 62.93 | 18.57 | 19.75 |
| *Boana pardalis* | MCNAM1836 | 64.54 | 18.01 | 21.13 |
| *Boana pardalis* | MCNAM4026 | 53.22 | 17.11 | 18.16 |
| *Boana pardalis* | MCNAM11583 | 57.66 | 15.58 | 17.35 |
| *Boana pardalis* | MCNAM11598 | 50.59 | 15.57 | 16.79 |
| *Boana pardalis* | MCNAM13532 | 49.83 | 14.71 | 16.76 |
| *Boana pardalis* | MCNAM13544 | 57.16 | 16.93 | 18.82 |
| *Boana pardalis* | MCNAM14690 | 54.70 | 15.74 | 17.01 |
| *Boana pardalis* | MCNAM14891 | 55.22 | 15.70 | 16.71 |
| *Boana pardalis* | MCNAM19022 | 59.49 | 17.15 | 18.95 |
| *Boana polytaenia* | MCNAM403 | 24.24 | 6.99 | 7.65 |

| | | | | |
|---|---|---|---|---|
| *Boana pombali* | MCNAM15858 | 50.45 | 16.90 | 15.79 |
| *Boana rosenbergi* | DZSJRP548 | 80.29 | 22.31 | 23.50 |
| *Boana xerophylla* | MNRJ92500 | 56.57 | 15.93 | 17.69 |
| *Boana xerophylla* | MNRJ92501 | 48.71 | 14.65 | 17.04 |

**Table S2:** Parameters used in the CT scanning process of the specimens. Legend: V - Voltage; A - Amperage; VS - Voxel Size.

| Specimen | V (kV) | A (µA) | VS (µm) | Projections | Frames |
|---|---|---|---|---|---|
| *Boana albomarginata* | | | | | |
| MCNAM2230 | 40 | 200 | 36.499997 | 1845 | 5 |
| MCNAM3750 | 50 | 240 | 34.200829 | 1920 | 5 |
| MCNAM11223 | 40 | 220 | 33.501667 | 1858 | 5 |
| MCNAM13607 | 40 | 220 | 25.999120 | 1857 | 5 |
| MCNAM13915 | 50 | 240 | 32.000261 | 1931 | 5 |
| MCNAM14696 | 50 | 240 | 34.998591 | 1943 | 5 |
| MCNAM15841 | 50 | 240 | 34.501110 | 1885 | 5 |
| MCNAM17675 | 40 | 220 | 30.001375 | 1839 | 5 |
| MCNAM18974 | 40 | 220 | 30.498856 | 1811 | 5 |
| MCNAM20674 | 50 | 240 | 33.501667 | 1865 | 5 |
| *Boana crepitans* | | | | | |
| MCNAM173 | 50 | 160 | 40.999733 | 1919 | 5 |
| MCNAM1063 | 50 | 240 | 38.001403 | 1940 | 5 |
| MCNAM1493 | 50 | 240 | 39.498327 | 1929 | 5 |
| MCNAM6384 | 50 | 240 | 41.501695 | 1914 | 5 |
| MCNAM7938 | 50 | 240 | 42.501138 | 1927 | 5 |
| MCNAM14889 | 50 | 220 | 44.500025 | 1835 | 5 |
| MCNAM18471 | 40 | 220 | 33.999148 | 1898 | 5 |

| | | | | | |
|---|---|---|---|---|---|
| MCNAM21084 | 50 | 220 | 39.198046 | 1872 | 5 |
| *Boana exastis* | | | | | |
| MNRJ35324 | 65 | 230 | 42.998619 | 2781 | 5 |
| MNRJ35325 | 65 | 230 | 42.998619 | 2958 | 5 |
| *Boana faber* | | | | | |
| MCNAM057 | 65 | 230 | 41.501695 | 2886 | 5 |
| MCNAM1018 | 65 | 230 | 45.499468 | 2592 | 5 |
| MCNAM6275 | 65 | 230 | 45.001988 | 2665 | 5 |
| MCNAM9510 | 50 | 240 | 51.774000 | 1943 | 5 |
| MCNAM10627 | 55 | 240 | 45.001988 | 1848 | 5 |
| MCNAM10762 | 65 | 230 | 45.001988 | 2572 | 5 |
| MCNAM16881 | 55 | 240 | 51.774000 | 1927 | 5 |
| MCNAM17006 | 65 | 230 | 41.999176 | 1202 | 5 |
| MCNAM17593 | 65 | 230 | 46.001431 | 1207 | 5 |
| MCNAM17595 | 50 | 200 | 24.999677 | 1606 | 5 |
| *Boana lundii* | | | | | |
| MCNAM1617 | 50 | 220 | 36.298316 | 1869 | 5 |
| MCNAM4112 | 50 | 220 | 35.998034 | 1810 | 5 |
| MCNAM6710 | 50 | 220 | 44.500025 | 1800 | 5 |
| MCNAM7129 | 50 | 220 | 40.000289 | 1818 | 5 |
| MCNAM7780 | 50 | 220 | 35.998034 | 1814 | 5 |
| MCNAM8007 | 50 | 220 | 43.998062 | 1817 | 5 |

| | | | | | |
|---|---|---|---|---|---|
| MCNAM8606 | 50 | 220 | 42.501138 | 1897 | 5 |
| MCNAM8800 | 50 | 220 | 45.001988 | 1872 | 5 |
| MCNAM9378 | 50 | 220 | 44.500025 | 1814 | 5 |
| MCNAM9389 | 50 | 220 | 46.001431 | 1808 | 5 |
| MCNAM9395 | 50 | 220 | 42.998619 | 1849 | 5 |
| MCNAM9513 | 50 | 220 | 42.998619 | 1840 | 5 |
| MCNAM9543 | 50 | 220 | 34.501110 | 1888 | 5 |
| MCNAM11894 | 50 | 220 | 42.501138 | 1821 | 5 |
| MCNAM12952 | 50 | 220 | 47.498355 | 1834 | 5 |
| MCNAM13010 | 50 | 220 | 40.000289 | 1759 | 5 |
| MCNAM14017 | 50 | 220 | 50.998647 | 1841 | 5 |
| MCNAM16133 | 50 | 220 | 40.000289 | 1880 | 5 |
| *Boana pardalis* | | | | | |
| MCNAM7931 | 50 | 240 | 42.501138 | 1862 | 5 |
| MCNAM1836 | 40 | 200 | 41.999176 | 1865 | 5 |
| MCNAM4026 | 50 | 240 | 33.501667 | 1912 | 5 |
| MCNAM11583 | 50 | 220 | 37.001960 | 1819 | 5 |
| MCNAM11598 | 50 | 240 | 38.498884 | 1874 | 5 |
| MCNAM13532 | 40 | 220 | 34.501110 | 1856 | 5 |
| MCNAM13544 | 50 | 240 | 40.000289 | 1916 | 5 |
| MCNAM14690 | 50 | 220 | 37.001960 | 1878 | 5 |
| MCNAM14891 | 50 | 240 | 40.000289 | 1922 | 5 |

| | | | | | |
|---|---|---|---|---|---|
| MCNAM19022 | 50 | 220 | 38.498884 | 1892 | 5 |
| *Boana polytaenia* | | | | | |
| MCNAM403 | 30 | 225 | 16.99965 | 1814 | 5 |
| *Boana pombali* | | | | | |
| MCNAM15858 | 50 | 240 | 37.49944 | 1768 | 5 |
| *Boana rosenbergi* | | | | | |
| DZSJRP548 | 65 | 230 | 43.99806 | 2711 | 5 |
| *Boana xerophylla* | | | | | |
| MNRJ92500 | 50 | 240 | 37.001960 | 1900 | 5 |
| MNRJ92501 | 50 | 240 | 32.999705 | 1876 | 5 |

**Table S3:** Analysis of Variance (ANOVA) for the dorsal view of the skull of species from the *Boana faber* clade, considering the difference, in Procrustes coordinates, between two photographs of the same individual and two digitization of anatomical landmarks in the same photograph. Legend: SQ - sum of squares; MQ - mean of squares; df - degree of freedom; F - F statistics; * - significant values (< 0.05).

| Effect | SQ | MQ | df | F | P (param.) |
|---|---|---|---|---|---|
| **Individual** | 0.43963188 | 0.0003330545 | 1320 | 13.33 | <0.0001* |
| **Side** | 0.00828664 | 0.0003452765 | 24 | 13.61 | <0.0001* |
| **Ind * Side** | 0.03348451 | 0.0000253671 | 1320 | 1.81 | <0.0001* |
| **Error 1** | 0.03769264 | 0.0000140226 | 2688 | 5.60 | <0.0001* |
| **Residual** | 0.01345429 | 0.0000025027 | 5376 | - | - |

**Table S4:** MANOVA considering the logarithm of the centroid size, species and the interaction between these parameters for the skull shape of species from the *Boana faber* clade (*Boana albomarginata*; *B. crepitans*; *B. faber*; *B. lundii*; *B. pardalis*). Legend: df, degree of freedom; SQ, sum of squares; MQ, mean of squares; RQ, R squared coefficient; F, F statistic; Z, size effect; Pr, P value; *, significant values (<0.05).

| | df | SQ | MQ | RSQ | F | Z | Pr (>SS) |
|---|---|---|---|---|---|---|---|
| **Centroid** | 1 | 0.001715 | 0.0017153 | 0.02883 | 2.4070 | 1.8384 | 0.031* |
| **Species** | 4 | 0.022193 | 0.0055482 | 0.37298 | 7.7855 | 6.5386 | 0.001* |
| **Centroid*Species** | 4 | 0.002811 | 0.0007029 | 0.04725 | 0.9863 | 0.0772 | 0.463 |
| **Residuals** | 46 | 0.032781 | 0.0007126 | 0.55094 | - | - | - |
| **Total** | 55 | 0.059500 | - | - | - | - | - |

**Table S5:** Pairwise test comparing the ontogenetic trajectory of skull shape of species from the *Boana faber* clade (*Boana albomarginata*; *B. crepitans*; *B. faber*; *B. lundii*; *B. pardalis*). Legend: d, distance between trajectories; UCL (95%), confidence interval between trajectory differences; Z, size effect; Pr > d, P value.

| | **d** | **UCL (95%)** | **Z** | **Pr > d** |
|---|---|---|---|---|
| *B. albomarginata*:*B. crepitans* | 0.09735456 | 0.10026793 | 1.50622516 | 0.067 |
| *B. albomarginata*:*B. faber* | 0.08506675 | 0.10777427 | 0.65297894 | 0.266 |
| *B. albomarginata*:*B. lundii* | 0.09047088 | 0.09412424 | 1.51941778 | 0.065 |
| *B. albomarginata*:*B. pardalis* | 0.08731880 | 0.13454334 | 0.05413966 | 0.460 |
| *B. crepitans*:*B. faber* | 0.05448181 | 0.09574206 | -0.40288394 | 0.650 |
| *B. crepitans*:*B. lundii* | 0.03396872 | 0.07612170 | -1.17809556 | 0.875 |
| *B. crepitans*:*B. pardalis* | 0.10323939 | 0.12795869 | 0.92885511 | 0.176 |
| *B. faber*:*B. lundii* | 0.04066470 | 0.09049704 | -1.13926165 | 0.858 |
| *B. faber*:*B. pardalis* | 0.06841940 | 0.13270252 | -0.70064106 | 0.763 |
| *B. lundii*:*B. pardalis* | 0.08986976 | 0.12251450 | 0.61363758 | 0.275 |

**Table S6:** Mahalanobis distances between groups.

| | *Boana albomarginata* | *Boana crepitans* | *Boana faber* | *Boana lundii* |
|---|---|---|---|---|
| *Boana crepitans* | 9.4828 | - | - | - |
| *Boana faber* | 9.6400 | 10.6795 | - | - |
| *Boana lundii* | 10.1442 | 6.2777 | 9.7739 | - |
| *Boana pardalis* | 8.4559 | 7.4730 | 9.2450 | 6.2628 |

**Table S7:** P values for permutation tests (10,000 permutations) for Mahalanobis distances between groups.

| | *Boana albomarginata* | *Boana crepitans* | *Boana faber* | *Boana lundii* |
|---|---|---|---|---|
| *Boana crepitans* | < 0.0001 | - | - | - |
| *Boana faber* | < 0.0001 | < 0.0001 | - | - |
| *Boana lundii* | < 0.0001 | < 0.0001 | < 0.0001 | - |
| *Boana pardalis* | < 0.0001 | < 0.0001 | < 0.0001 | < 0.0001 |

**TableS8:** Procrustes distance between groups.

| | *Boana albomarginata* | *Boana crepitans* | *Boana faber* | *Boana lundii* |
|---|---|---|---|---|
| *Boana crepitans* | 0.0437 | - | - | - |
| *Boana faber* | 0.0456 | 0.0496 | - | - |
| *Boana lundii* | 0.0486 | 0.0386 | 0.0380 | - |
| *Boana pardalis* | 0.0514 | 0.0463 | 0.0758 | 0.0628 |

**Table S9:** P values for permutation tests (10,000 permutations) for Procrustes distances between groups.

| | *Boana albomarginata* | *Boana crepitans* | *Boana faber* | *Boana lundii* |
|---|---|---|---|---|
| *Boana crepitans* | < 0.0001 | - | - | - |
| *Boana faber* | < 0.0001 | <0.0002 | - | - |
| *Boana lundii* | < 0.0001 | < 0.0001 | < 0.0001 | - |
| *Boana pardalis* | < 0.0001 | < 0.0001 | < 0.0001 | < 0.0001 |
